# Reducing pain in children with cancer at home: a feasibility study of the KLIK pain monitor app

**DOI:** 10.1007/s00520-021-06357-9

**Published:** 2021-06-16

**Authors:** Julia D. H. P. Simon, Sasja A. Schepers, Martha A. Grootenhuis, Maarten Mensink, Angelique D. Huitema, Wim J. E. Tissing, Erna M. C. Michiels

**Affiliations:** 1 Princess Máxima Center for Pediatric Oncology, Heidelberglaan 25, 3584 CS Utrecht, The Netherlands; 2 Department of Pediatric Oncology, University of Groningen, University Medical Center Groningen, Groningen, The Netherlands

**Keywords:** Pain, Pediatric Oncology, mHealth/eHealth, Feasibility, Implementation

## Abstract

**Purpose:**

This study assessed adherence to, feasibility of, and barriers and facilitators of implementation of an app developed to monitor and follow-up with pain in children with cancer at home.

**Methods:**

Children (8–18 years) receiving cancer treatment (all diagnoses) or their parents (of children aged 0–7 years) used the KLIK Pain Monitor app for 3 weeks. Pain was assessed twice daily using an 11-point numeric rating scale (NRS-11) (ranging from 0 to 10). Healthcare professionals (HCP’s) from the hospital’s Pediatric Pain Service were instructed to follow-up with clinically significant pain scores (≥ 4) within 120 min (scores 4–6) or 30 min (scores 7–10). Adherence, feasibility, and implementation outcomes were assessed using questionnaires, app log data, and interviews.

**Results:**

Twenty-seven children (*M* age = 7.3 years, 51.8% male) and six HCP’s participated. Sixty-three percent (*N* = 17) of families used the app on a daily basis during three weeks, and 18.5% (*N* = 5) reported pain scores twice daily during that time (*family adherence*). Twelve out of 27 children (44.4%) reported a clinically significant pain score at least once. In 70% (14/20) of clinically significant pain scores, HCP’s followed-up with families within the set timeframe (*HCP adherence*). Outcomes reveal feasibility for the majority of app functions (i.e., positive evaluation by ≥ 70% families/HCP’s), and non-feasible aspects could be resolved. Identified barriers and facilitators were used to improve future implementation efforts.

**Conclusion:**

Use of the KLIK Pain Monitor app seems feasible. Future research will determine its effectiveness in reducing pain in children with cancer at home.

## Introduction

Pain is a common and disconcerting symptom during all stages of childhood cancer with prevalence rates varying between 40 and 78% [1–5]. Changes in therapeutic regimens cause children to spend less time in the hospital and more time at home [6–10], making families increasingly responsible for the management of pain [6, 9]. Studies on pain management at home reveal parental misconceptions (e.g., pain is unavoidable during cancer) [1] and concerns regarding analgesic use (e.g., pain medication is addictive) [11]. A previous study in children (1–18 years old) receiving outpatient chemotherapy revealed that in one third of clinically significant pain incidents (score ≥ 4 on scale of 0–10 [12, 13]) occurring in the home setting, no analgesic medication was used [5]. It seems that despite the availability of effective pain interventions (either pharmacologic [14, 15] or nonpharmacologic [16–19] in nature) for children with cancer, parents tend to undertreat pain [3]. As pain has been related to poor quality of life, suffering and morbidity [20], combined with the notion that suffering from persistent pain during the treatment of cancer can extend into survivorship [21], it is imperative to address this problem.

Interventions for the home setting are warranted. Some efforts have already been made to address this using mHealth, such as the Pain Squad + smartphone app [22], the tablet-based Pain Buddy program [23], and the Color me Healthy app [24], which were all developed to improve pain management in children with cancer. The KLIK Pain Monitor app, named after the existing KLIK PROM (patient-reported outcome measures) portal [25], was developed to reduce pain in children aged 0–18 years old at home during cancer treatment with the aim to (1) monitor pain in the home setting, enabling healthcare professionals (HCP’s) to follow-up with families and offer help more quickly, and (2) provide families with psycho-educational information about pain. A future goal is to integrate data from the app into the KLIK PROM portal, which has already been implemented at the hospital. The distinction between the KLIK Pain Monitor app and most previously developed mHealth-initiatives lies in its target user (i.e., all children with cancer versus 6/8–18 year olds). By creating an app that bridges the gap between the hospital and home setting, we aim to improve pain management and decrease pain in children with cancer.

Stakeholder (i.e., end user) involvement is a prerequisite for the development of purposeful mHealth interventions fit for effective use in practice [26]. This study therefore aims to assess user adherence to, feasibility of, and barriers/facilitators of implementation based on family and HCP experiences with the app. Outcomes will be used to improve the app and processes involved.

## Methods

### The KLIK Pain Monitor app

**Fig. 1:**
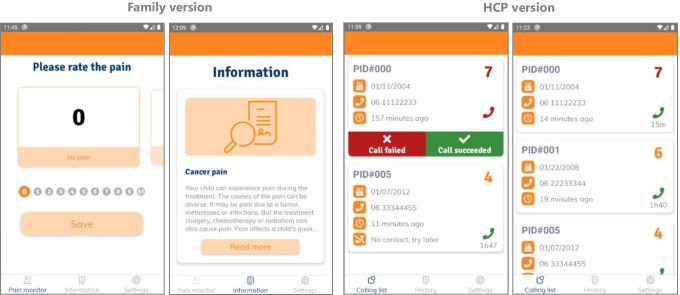
Family and HCP version of the KLIK Pain Monitor app (English translation)

**Fig. 2:**
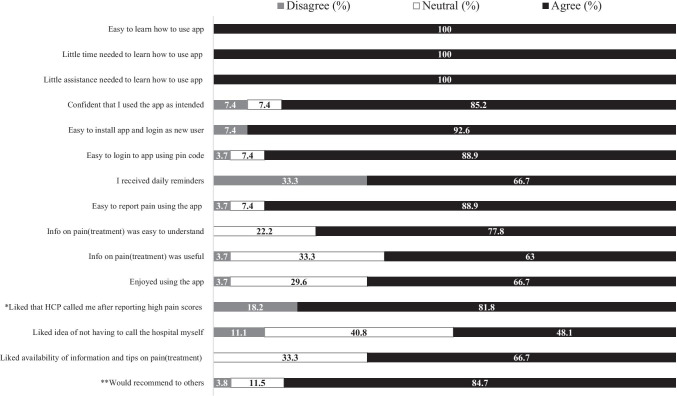
Feasibility (usability, learnability, desirability) rated by families. *Note.* *N = *27 families. Responses rated on five-point Likert-scale divided into categories of disagree (scores 1, 2), neutral (score 3) and agree (scores 4, 5). *N* = 11 for item with * ( only families called by HCP during study included); *N* = 26 for item with ** (one missing)

**Fig. 3:**
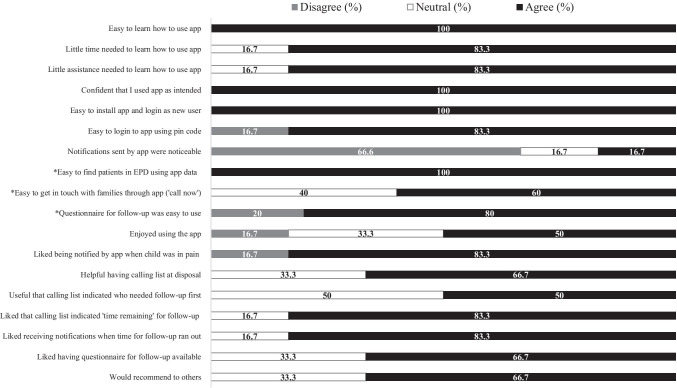
Feasibility (usability, learnability, desirability) rated by HCP’s. *Note.* *N = *6 HCP's. Responses rated on five-point Likert-scale divided into categories of disagree (scores 1, 2), neutral (score 3) and agree (scores 4, 5). *N* = 5 for items with * ( only HCP who called by families during study included)

The KLIK Pain Monitor app for Apple and Android was commissioned by the Princess Máxima Center for Pediatric Oncology and the software was developed according to secure and controlled processes (ISO72001/NEN75010 approved Information Security Management System) by an external web design company (Biomedia). To ensure user privacy, the app uses two-factor authentication (2FA) login, and a Data Protection Impact Assessment (DPIA) was carried out and approved by the hospital’s Data Protection Officer. Currently, there are three versions of the app: a parent version (for kids aged 0–7 years old), a child version (for kids aged 8–18 years old, for which language was adapted and approved by the Dutch children’s cancer association) and a HCP version. Screenshots of the translated parent/child and HCP version of the app can be found in Figs. 1, 2 and 3. The parent/child version of the app featured psycho-educational information about pain, medication and nonpharmacologic interventions suitable to the home setting. This information was composed by a medical psychologist, pediatric oncologist specialized in palliative care, and a representative of the center’s Pain Service, based on the WHO Guidelines on the Pharmacological Treatment of Persisting Pain in Children with Medical Illnesses [27], the Dutch Pediatrics Association Guideline on the treatment of pain [28], and a clinical practice guideline on the pharmacological and psychological management of pain in children with cancer [17].

The family version of the app allowed children (aged 8–18) or parents (children aged 0–7) to report pain intensity at the time on an 11-point numeric rating scale (NRS-11) ranging from 0 (no pain) to 10 (worst pain imaginable). When a score ranging between 0 and 3 was reported, families were redirected to interventions suitable to the home setting on the psycho-educational information page of the app. When a clinically significant pain score was reported (≥ 4), a notification was forwarded to the family (stating the time in which they would be contacted, and instructions to contact the hospital themselves in acute situations requiring immediate follow-up), as well as the calling list of the HCP version of the app. They were instructed to call the family within a set time frame (i.e., within 2 h for pain scores 4–6 and within 30 min for scores 7–10). In the HCP calling list, the reported pain intensity score, remaining time for follow-up (based on set time frame), a patient identification number (PID), date of birth, and a phone number provided by the family for the purposes of this study were visible. The attending HCP would use the PID (and date of birth as cross check) to find the patient in the hospital’s electronic patient dossier to read up on essential medical background information, before calling the family. During follow-up, HCP’s were instructed to use a standardized questionnaire and decision tree for pain management based on the center’s Pediatric Pain Service standard of care.

### Procedure and participants

This study included children aged 0–18 years old (all diagnoses) receiving chemotherapy at the Princess Máxima Center for Pediatric Oncology in Utrecht, The Netherlands. In this national center, all care for children with cancer is centralized. Participants needed to have a sufficient understanding of the Dutch language to participate. Finally, as the app was developed to decrease pain in children in the home setting, children needed to be home at the time of the study (i.e., not hospitalized). Participating HCP’s consisted of members of the hospital’s Pediatric Pain Service (*N* = 4 nurses specialized in pediatric pain treatment), and two pediatric oncologists. Approval for the study was obtained from the Internal Review Board of the hospital.

Families of eligible children received both oral and written information about the study. If a family agreed to participate, an informed consent form was signed. The coordinating researcher offered support with downloading the app, after which families received their login information via email. For children aged 0–7 years old, a parent was asked to use the app and report pain scores based on their evaluation of the child’s pain (i.e*. parent proxy-reporting*) [29, 30]. Children aged 8–18 years old were assumed capable to scoring self-reports of pain intensity using the NRS-11 scale [31]. However, not all children in this age group owned a smartphone or were capable of using the app independently. Thus, parents were allowed to help (i.e., using their phone and offering support with pain reporting). Still, the focus for this age group was *self-reported* pain (child’s self-assessment of the pain). Families were clearly instructed that whoever used the app (child/parent) during the study, should also complete the evaluative questionnaire and interview at the end of the study. Thus, if a parent assisted in using the app, they also assisted in completing the evaluative questionnaire and were present during the interview.

Families were asked to use the KLIK Pain Monitor for three consecutive weeks and report pain at least twice daily (morning and evening), and whenever deemed necessary (ad hoc). Daily reminders were sent in the morning and evening, for which families were able to set the exact times. The minimum requirement of two pain assessments per day created the opportunity to test all functionalities of the app.

Adherence was assessed using log data from the app. All app-users (families and HCP’s) completed a feasibility questionnaire after completion of the study. To identify barriers of, and facilitators to future implementation, app-users were interviewed (semi-structured) about their experiences with the app.

### Measures

This study used a mixed-method design, consisting of quantitative (standardized questionnaires and log data from the app) as well as qualitative (semi-structured interviews) methods.

#### Background and medical characteristics

The child’s age, sex, and medical characteristics (diagnosis, time since diagnosis, stage/risk levels, and treatment modalities (surgery, chemotherapy, radiation, transplant)) were obtained from the medical chart. Medical characteristics were used to complete the Intensity of Treatment Rating (ITR-3) [32]. Intensity levels were as follows: level 1 (least intensity), 2 (moderately intensive), 3 (very intensive), and 4 (most intensive). The ITR-3 was completed individually by two pediatric oncologists (WT and EM), after which scores were discussed and consensus was reached on the intensity level of treatment of each patient.

#### Adherence

Adherence reflected the extent to which families and HCP’s were able to use the app as intended [33]. For families, this meant reporting pain scores at least twice daily for 3 weeks. If patients were admitted to the hospital during this period, families were asked to stop using the app temporarily and resume once they returned home. For HCP’s, adherence related to responding to clinically significant pain scores within the defined time range: 120 min for scores 4–6, and 30 min for scores 7–10. Adherence to the app was assessed using log data obtained through the app server.

#### Feasibility

Feasibility was assessed using a questionnaire (separate version for families and HCP’s) with statements regarding app functions. The questionnaire was adapted from Hochstenbach et al. (2016) [34] and two versions were composed: for families and for HCP’s. The questionnaires focused on learnability (*N* = 4 items), usability (*N* = 6 items), and desirability (*N* = 4 (families), *N* = 7 (HCP’s)) of the app. Learnability reflected the time and effort required for families and HCP’s to learn how to use the application as intended (e.g., “*It was easy to learn how to use the app*”). Usability reflected the extent to which families and HCP’s could use the app with effectiveness, efficiency and satisfaction (e.g., “*The information provided by the app on pain (treatment) was easy to understand*”). Desirability reflected the extent to which the application was pleasant and engaging to use [34] (e.g., “*I liked that HCP’s called me when I reported high pain scores*”). The questionnaire contained an additional item to assess whether users would recommend the app to others. App-users rated their agreement with these statements on a 5-point Likert scale (1 = strongly disagree, 2 = disagree, 3 = undecided, 4 = agree, 5 = strongly agree). Higher scores indicated better learnability, usability, and desirability. Internal consistency was evaluated. Cronbach’s alphas for the family version were 0.54 (learnability scale), 0.40 (usability scale), and 0.79 (desirability scale). For the HCP version, these were 0.84 (learnability scale), 0.60 (usability scale), and 0.80 (desirability scale).

#### Barriers and facilitators of implementation

Interviews were carried out with all app-users (families and HCP’s). Whoever used the app (parent or child) was interviewed. A semi-structured interview guide was composed and focused on three main themes: use and general satisfaction with the app, technical functioning of the app, and supportiveness of the app regarding pain management.

### Analytic strategy

#### Adherence

In order to determine adherence to the *KLIK Pain Monitor app*, descriptives were used to assess the percentage of patients that reported scores twice daily in the home setting for 3 weeks (i.e., 21 days) (*family adherence*), and the percentage of incidences in which healthcare professionals called within the set time range when clinical scores were reported by families (*HCP adherence*). The threshold was reached if at least 70% of families/HCP’s adhered to app use as intended. If adherence was below that cut-off point, the process involved was re-evaluated and measures were taken to make improvements.

#### Feasibility

We assessed responses on the feasibility questionnaire for families and HCP’s separately. A statement (each relating to specific app functions) was found feasible if it was rated with a 4 (agree) or higher by at least 70% of families/HCP’s. Conversely, if a statement was rated with a 2 (disagree) or lower by at least 30% of families/HCP’s, the corresponding app-function was closely re-evaluated and measures were taken to make improvements.

#### Barriers and facilitators of implementation

Transcripts of the interviews were made and all interviews were audio recorded. Transcripts were then thoroughly read and thematic analysis was performed by the interviewer (JS) to identify recurring topics and meaningful themes within the data [35, 36]. The following main themes emerged: technical functioning, user friendliness, content and functionalities, and impact on pain care. Subsequently, the transcripts were analyzed by two researchers (JS and SS) independently to identify barriers and facilitators for future implementation. These were categorized into either one of the main themes. Afterwards, the researchers discussed their findings during several meetings and consensus was reached on which barriers and facilitators were mentioned. To identify relevance of specific topics, a list was composed with all identified barriers and facilitators, and how often they were mentioned. If a barrier or facilitator was mentioned by at least 30% of families/HCP’s, it was marked “relevant” by the researchers. For relevant facilitators, efforts were made to reinforce their impact on successful future implementation; for relevant barriers, measures were taken to prevent their impact on successful future implementation.

## Results

Forty-one families of children with cancer were invited to participate in the study. Of those, 28 families agreed to participate and signed informed consent (response rate: 68%). No families were ineligible for participation due to a lack of devices. The most common motivation for non-participation was the absence of pain at the time of intended inclusion. One family dropped out after signing informed consent but before they started using the app due to bad timing with regard to the child’s treatment (feeling overwhelmed). The characteristics of the remaining 27 children are summarized in Table 1, as well as HCP characteristics.

**Table 1:** Child and HCP characteristics *n* (%)

Child characteristics (*N* = 27)	
Mean child age (years (SD), range)	7.33 (5.00), 1–17
Child sex (male)	14 (51.9)
Diagnosis category	
Leukemia/lymphoma	18 (66.7)
Brain/CNS tumors	4 (14.8)
Solid tumors (non-CNS)	5 (18.5)
Time since diagnosis	
< 3 months	5 (18.5)
< 4 to 6 months	3 (11.1)
6–11 months	7 (25.9)
1–2 years	12 (44.4)
Intensity of treatment rating (ITR)	
1 (least intensive)	0 (0.0)
2 (moderately intensive)	20 (74.1)
3 (very intensive)	4 (14.8)
4 (most intensive)	3 (11.1)
HCP characteristics (*N* = 6)	
Mean HCP age (years (SD), range)	48.2 (9), 35–57
HCP sex (male)	1 (16.6)
Mean work experience (years (SD), range)	9.42 (10), 0.5–27

Note. *HCP *Healthcare professional; *SD *standard deviation; *n *individuals in each category; *CNS *Central Nervous System

Of the participating children, eight children (29.7%) used the app themselves and two children (7.4%) used the app with the help of a parent. For the remaining children, the app was used by a parent (mothers: *N* = 12, 44.4%; fathers: *N* = 2, 7.4%, both parents: *N* = 3, 11.1%).

### Adherence

#### Families

Log data from the app shows that 63% (*N* = 17) of families used the app at home on a daily basis during the three study weeks, and 37% (*N* = 10) used the app for a shorter period (*minimum number of days* = *7*). Of all families, 18.5% (*N* = 5) used the app at home for three weeks and reported pain scores twice daily during that time (*family adherence*).

Of the total of 976 reported NRS-11 pain scores, twenty clinically significant pain scores were reported by 12 families. Thus, 44.4% (12/27) of families reported a clinically significant pain score at least once. Of the clinically significant reported pain scores, 50% (*N* = 10) occurred during the nights/evenings/weekends, and 50% (*N* = 10) on working days between 8 a.m.–5 p.m.

#### HCP’s

In 70% (14/20) of clinically significant incidences, HCP’s called families within the set timeframe (HCP adherence).

### Feasibility

#### Families

The majority of statements (9/15) were rated with a 4 (agree) or higher by at least 70% of families and were found feasible (Fig. 1). One statement (“*I received daily reminders at the times I chose*”) was rated with a 2 (disagree) or lower by at least 30% of families, and was found not feasible. The remaining statements did not reach the cut-off for feasibility nor non-feasibility due to neutral responses (score 3).

#### HCP’s

The majority of statements (14/18) were rated with a 4 (agree) or higher by at least 70% of HCP’s (Fig. 2) and were found feasible. One statement (*“Pop-ups (reminders, notifications) sent by the app were noticeable”*) was rated with a 2 (disagree) or lower by at least 30% of HCP’s, and was found not feasible. The remaining statements did not reach the cut-off for feasibility nor non-feasibility due to neutral responses (score 3).

The feasibility questionnaire included one added item assessing whether users would recommend the app to others. Of the families, 81.5% said that they would recommend the app to other children/parents, and 66.7% of HCP’s said that they would recommend the app to other HCP’s.

### Barriers and facilitators of implementation

#### Families

**Fig. 4:**
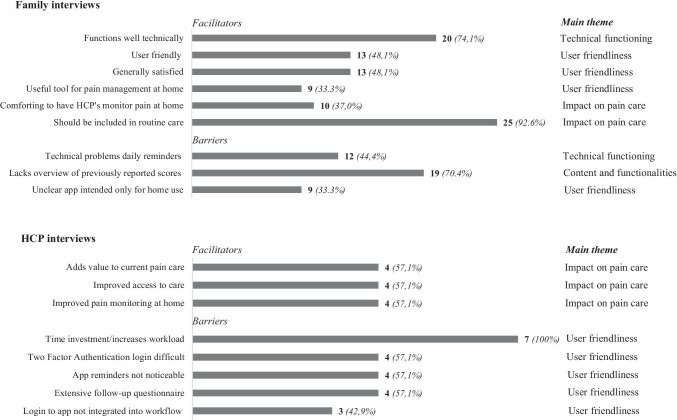
Facilitators and barriers identified in family and HCP interviews. Note. *N* =  27 families and *N* = 7 HCP's. Facilitator/barrier included if mentioned by at least 30% of families/HCP's

Results of the interviews can be found in Fig. 4. Based on the interviews with families, six relevant facilitators and three relevant barriers were identified (i.e., mentioned by at least 30% of families). The facilitators related to technical functioning (*“It worked perfectly: customer friendly, intuitively, simple. I didn’t experience any problems”*), impact on pain care (“*We don’t want to call the hospital all the time. With the app, you get the sense that pain is being monitored and they call us when we report high pain scores. That is very comforting. It gives you the sense that you’re being taken care of*”), and user friendliness of the app (*“It was really easy and clear how to use the app”)*. The identified barriers related to technical problems with daily reminders (*“I didn’t always receive the daily reminders. At one point, I didn’t receive them for two days”*), content and functionalities (*“The only thing that was missing, was an overview of previously reported scores. That way, you get a sense of patterns of pain”*), and user friendliness *(“It wasn’t immediately clear to us that the app was only meant for use at home and not during hospitalization”*).

#### HCP’s

Based on the interviews with HCP’s, three relevant facilitators and five relevant barriers have been identified (i.e., mentioned by at least 30% of HCP’s). Facilitators mainly related to impact on pain care (“*I think that the app will increase our knowledge on how often kids are in pain at home. And it enables us to provide them with care much quicker”*). The identified barriers related to user friendliness of the app (“*If we start using the app there will be extra shifts, extra workload*”).

## Discussion

This study aimed to assess adherence to, feasibility of, and barriers and facilitators of implementation of a newly developed app to reduce pain in children with cancer at home, by providing HCP’s and families with a tool for real-time feedback and educational information about pain (management).

Family adherence (i.e., reporting pain scores twice daily for three weeks) was 18.5%. Log data from the app reveals that families did not receive daily reminders for pain reporting during one-third of the study period. This was the only adjustment made to the app over the course of the study. A consecutive study will closely monitor the effects of the specific adaptations made to the app and processes involved as a result of this feasibility study. During the month the technical bug was active, zero family adhered to our request of reporting pain twice daily for 3 weeks. After the bug had been resolved, family adherence increased to 38.4%, indicating that the bug affected family adherence. However, 38.4% is still low compared to another study with a similar app and patient population (children with cancer aged 8–18 years old), in which adherence to pain assessment was 62% [37]. Another possible explanation for low family adherence in the current study might be a relatively low prevalence of pain in the study sample. Of the total of 976 reported pain scores, 20 scores were clinically significant (2%). This is in shrill contrast with the 78% pain prevalence found in children with cancer in a previous study [5]. Thus, adherence and prevalence will be closely monitored in consecutive studies.

For HCP’s, adherence (i.e., follow-up with families within the set timeframe) was 70%, reaching the pre-defined threshold for adherence. However, as feasibility questionnaires as well as interviews reveal that HCP’s did not always notice the notifications sent when a clinically significant score was reported, we believe profit can still be made. Thus, the notifications have been assigned a more distinctive sound and the effect on HCP adherence will be assessed in consecutive studies.

The cut-off for feasibility (learnability, usability, desirability) was reached for the majority of app functionalities and non-feasible functions have been addressed (i.e., technical bug daily reminders, non- distinctive sound of HCP notifications). Generally, families (81.5%) and HCP’s (66.7%) said they would recommend the app to others.

Barriers and facilitators mentioned in the interviews have been taken into account as well. Some families said the app should have an overview of previously reported scores. This was not one of the original aims of the app, but it has been added to the list of possible future functionalities. Also, since it was not clear to all families that the app was only meant for use at home, a flyer with clear instructions was developed to hand out to families in consecutive studies and future implementation. Facilitators mentioned by families related to improved care for patients and user friendliness of the app.

Barriers identified in HCP interviews mainly related to time consumption and increased workload. The list of questions used by HCP’s as a guideline for follow-up with patients (based on the hospital’s Pediatric Pain Service standard of care) was rated ‘too extensive’ and has since then been reviewed by the Pediatric Pain Service. HCP’s indicated that the login process was not yet integrated into their workflow and could be easily forgotten. This is something we cannot resolve immediately, and we think that this is a matter of time to get used to. With regard to the 2-FAlogin process (“time consuming”), as this is a privacy requirement we were unable to make alterations. Facilitators mainly related to improved care for patients, and user friendliness of the app. Thus, although HCP’s are generally positive about use of the app and its potential benefits for patient care, their worries relating to workload need to be addressed. Worries related to workload might also account for the fact that one-third of HCP’s indicated that they would not recommend the app to other HCP’s. In view of the fact that only a small number (*N* = 20, 2%) of reported scores required follow-up, it is possible that external factors have also influenced HCP’s attitudes towards working with the app. As the Princess Máxima Center is a relatively new hospital in which HCP’s are still getting used to new workflows and division of tasks, it is possible that HCP’s experience a resistance to (additional) change [38]. This will be addressed in consecutive studies.

These outcomes are in line with a previous study assessing barriers and facilitators of implementation of an online tool monitoring electronic patient-reported outcomes (KLIK) [25]. Similar to the current study, barriers mainly related to organizational context (i.e., time), whereas facilitators related to the intervention (i.e., simplicity of use) and outcome expectations (i.e., more efficient detection of problems). Addressing organizational aspects such as capacity, financial resources, and time is an essential prerequisite for the successful implementation of innovations [39], and will be taken into account in future efforts.

The current study has some limitations. Firstly, a technical bug caused the daily reminders for pain reporting not to be sent to families for a majority of the study period, affecting family adherence. However, it should be noted that the daily reminders were merely instituted to guarantee sufficient pain scores to test all functionalities of the app. As the final goal of the app is to provide families with a tool to report pain when necessary (not at set times), the daily reminders will be optional. Secondly, this study reflects the experiences of a small sample of children and parents, whose perspectives might not be representative for all children with cancer receiving treatment in the home setting. However, with regard to patient characteristics (age, gender, diagnosis, intensity of treatment), this group reflects a realistic cross section of the patient population. Thirdly, as the app is currently only available in Dutch, non-Dutch speaking families could not participate. Translation of the app to different languages is an important goal for the future. Fourthly, only families with access to a smartphone and access to the Internet were able to participate in this study. However, as The Netherlands is one of the leading European countries with regard to households with internet access (98%) and smartphones (87%) [40], we do not believe this has impacted the outcomes. And fifthly, not all sub scales of the feasibility questionnaires for families and HCP’s showed good internal consistency. However, as we wanted to analyze individual items to assess specific functionalities of the app, rather than calculate mean scores for sub scales, we see the questionnaire as a valid tool for this purpose.

We can conclude that patients, parents, and HCP’s are generally positive about the KLIK Pain Monitor and use of the app seems feasible for implementation at the Princess Máxima Center for Pediatric Oncology. This study is an important preliminary step in the implementation process, as tailoring interventions based on evaluation with stakeholders (patients, parents, HCP’s) will ultimately benefit effective use in practice [26]. As feasibility has been established, the next step is to assess effectiveness of the app in reducing pain in children at home, in a randomized controlled trial (RCT). If found effective, the KLIK Pain Monitor app will be implemented and function as a bridge between the hospital and home setting, improving pain management at home and decreasing pain in children with cancer.

## Data Availability

The data that support the findings of this study are available from the corresponding author upon reasonable request.

## Code availability

SPSS version 25.0 was used for all analyses.
